# Women's and peer supporters' experiences of an assets‐based peer support intervention for increasing breastfeeding initiation and continuation: A qualitative study

**DOI:** 10.1111/hex.13042

**Published:** 2020-03-21

**Authors:** Jenny Ingram, Gill Thomson, Debbie Johnson, Joanne L. Clarke, Heather Trickey, Pat Hoddinott, Stephan U. Dombrowski, Kate Jolly, the ABA Study Team

**Affiliations:** ^1^ Centre for Academic Child Health University of Bristol Bristol UK; ^2^ Maternal and Infant Nutrition and Nurture Unit (MAINN) University of Central Lancashire Preston UK; ^3^ Institute of Applied Health Research University of Birmingham Birmingham UK; ^4^ DECIPHER Department of Social Medicine Cardiff University Cardiff UK; ^5^ Nursing, Midwifery and Allied Health Professions Research Unit University of Stirling Stirling UK; ^6^ Faculty of Kinesiology University of New Brunswick Fredericton NB Canada; ^7^ Division of Psychology University of Stirling Stirling UK

**Keywords:** assets‐based approach, breastfeeding, infant feeding, peer support, qualitative interviews

## Abstract

**Background and context:**

Breastfeeding peer support is valued by women, but UK trials have not demonstrated efficacy. The ABA feasibility trial offered proactive peer support underpinned by behaviour change theory and an assets‐based approach to women having their first baby, regardless of feeding intention. This paper explores women's and infant feeding helpers' (IFHs) views of the different components of the ABA intervention.

**Setting and participants:**

Trained IFHs offered 50 women an antenatal meeting to discuss infant feeding and identify community assets in two English sites—one with a paid peer support service and the other volunteer‐led. Postnatally, daily contact was offered for the first 2 weeks, followed by less frequent contact until 5 months.

**Methods:**

Interviews with 21 women and focus groups/interviews with 13 IFHs were analysed using thematic and framework methods.

**Results:**

Five themes are reported highlighting that women talked positively about the antenatal meeting, mapping their network of support, receiving proactive contact from their IFH, keeping in touch using text messaging and access to local groups. The face‐to‐face antenatal visit facilitated regular text‐based communication both in pregnancy and in the early weeks after birth. Volunteer IFHs were supportive of and enthusiastic about the intervention, whereas some of the paid IFHs disliked some intervention components and struggled with the distances to travel to participants.

**Conclusions:**

This proactive community assets‐based approach with a woman‐centred focus was acceptable to women and IFHs and is a promising intervention warranting further research as to its effect on infant feeding outcomes.

## 1 BACKGROUND

Peer support is a method of delivering social support to others who share common experiences. Internationally, breastfeeding peer support interventions have been shown to have a significantly greater effect on any and exclusive breastfeeding in low‐ or middle‐income countries compared to high‐income countries.^1^ While UK randomized controlled trials of breastfeeding peer support have not demonstrated efficacy, policy recommends peer support for socially disadvantaged women.^1^, ^2^, ^3^, ^4^ Qualitative studies report that women value peer support and disparities in outcomes may be due to implementation and context.^5^, ^6^ Currently, there are a range of breastfeeding peer support programmes (both paid and volunteer) available in the UK. To increase acceptability, effectiveness and inclusiveness, programmes are recommended to be woman‐centred (including help with formula and mixed feeding), be offered proactively and focus on the early weeks.^6^, ^7^, ^8^, ^9^, ^10^, ^11^, ^12^

**Figure 1:**
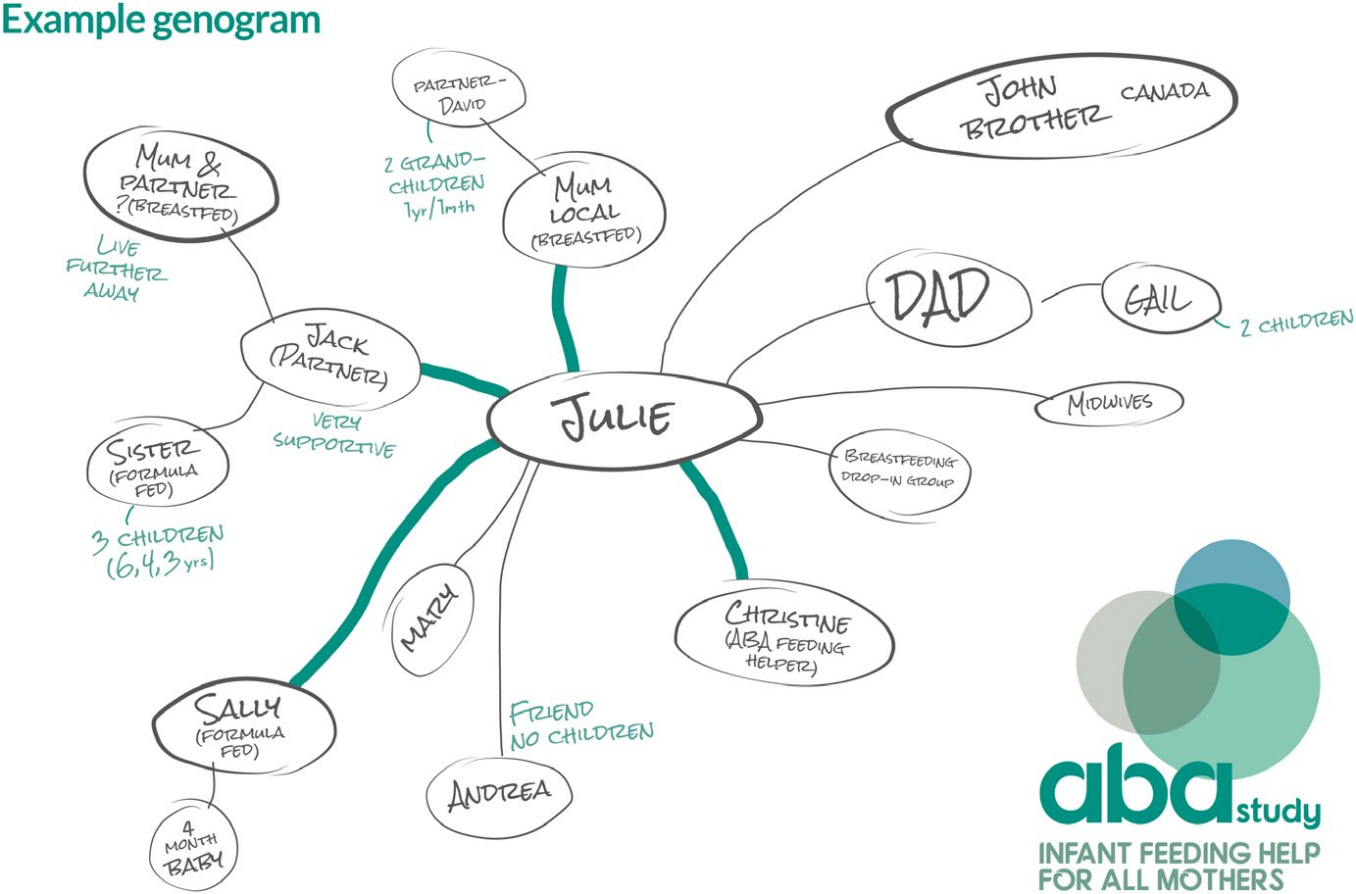
Mapping the friends and family tree (Infant Feeding Genogram)

The ABA (Assets‐based feeding help Before and After birth) intervention was developed and offered within a feasibility randomized controlled trial. It combined proactive peer support underpinned by behaviour change theory, particularly providing social support and restructuring the environment, (COM‐B model)^13^ with an assets‐based approach to women, regardless of their feeding intention.^14^ Assets‐based approaches focus on positive capabilities of individuals and communities, rather than their needs, deficits and problems.^15^ The ABA intervention was delivered by trained infant feeding helpers (IFHs) who offered women an antenatal meeting to discuss infant feeding and help to identify their community assets (including local groups) and used a conversational approach to develop a friends and family tree diagram of infant feeding experiences and potential support (Infant Feeding Genogram^16^ (see Figure 1)). Postnatally, daily contact was offered for the first two weeks after birth, followed by less frequent contact until five months as women wanted, through face‐to‐face contacts, phone calls and text messages.

The feasibility trial was successful in recruiting primiparous women, including those from areas of socioeconomic disadvantage, with adequate follow‐up rates; recruiting and training existing peer supporters to the new ABA role; and delivering the intervention with satisfactory fidelity; and it was acceptable to women, IFHs and maternity services.^17^ The proportion of ABA intervention women reporting breastfeeding initiation and any breastfeeding at 8 weeks and 6 months was consistently higher than in the usual care group.^17^ The aim of this paper was to understand the views of women and IFHs of the ABA intervention components when delivered by two different peer support services.

## 2 METHODS

### 2.1 Setting

The ABA feasibility trial was undertaken in two geographical sites in England. The sites were selected because they had contrasting volunteer and paid peer support services operating, in areas with high levels of socioeconomic disadvantage and low rates of breastfeeding initiation and continuation. Existing breastfeeding peer supporters (n = 13) at the two sites received six hours of ABA IFH training.^14^ At Site A, the ABA intervention was delivered by paid IFHs (n = 6) in an inner‐city setting; at Site B, the IFHs (n = 7) were volunteers in a more rural setting. As part of their existing job, IFHs in Site A generally worked in a more ethnically diverse area of the city some distance from our study site. In Site B, IFHs were volunteers at local neighbourhood breastfeeding groups. To deliver the antenatal session, Site B IFHs met women at local children's centres and cafes as they were not insured to visit women in their homes, whereas the paid workers in Site A were able to provide home visits.

### 2.2 Participants

Women, regardless of feeding intention, were recruited to the ABA trial between February and August 2017 through community midwifery clinics. Midwives provided women with study information, and then, a researcher approached women at antenatal clinics to gain informed consent. Overall, 103 primiparous women were recruited, 50 of whom received the ABA intervention.^14^ Semi‐structured qualitative interviews were undertaken with a sample of 21 women who received the intervention, who returned their 8‐week outcome questionnaire and who had agreed to be interviewed. Women with different ages, feeding experiences and levels of engagement with ABA were purposively selected and interviewed at home when their babies were aged 4‐21 weeks. These interviews explored their views and experiences of the ABA intervention and ranged from 45 to 90 minutes in duration. All 13 IFHs took part in one of two focus groups (n = 9—four at Site A and five at Site B) or a telephone interview (n = 4; two from each site). IFH focus groups/interviews were led by GT, who had no prior interactions with the IFHs. Other researchers (JI, JC, DJ) attended as note takers. The peer supporter co‐ordinator at Site B also attended the focus group to offer insights from her perspective. The focus groups/interviews explored experiences of the ABA intervention and its delivery; focus groups were ~100 minutes and interviews were ~30 minutes long.

### 2.3 Analysis

Data analysis was carried out by trained qualitative researchers (JC, DJ, GT, JI) who have extensive experience of qualitative research and evaluation of breastfeeding peer support services, from psychology, health services research and midwifery backgrounds. Interviews used a topic guide (see Appenidx S1), were audio‐recorded, transcribed verbatim, anonymized and imported into NVivo 11 (QSR International Pty Ltd) for coding. Transcripts were analysed using thematic methods by developing a coding framework and a series of themes to describe women's and IFHs' experiences.^18^ Subsequently, views of the women and IFHs were compared using framework analysis.^19^ A subset of four transcripts was independently coded by GT, JC and DJ, followed by discussions to agree the coding framework. This framework was used by two researchers (JC, DJ) to code the remaining transcripts, with ongoing discussions to consider and agree any changes as needed. All analytical decisions were shared with the wider research team using a consensus process to agree the final coding and thematic framework.

Patient and public involvement (PPI) was essential in shaping the development of the ABA study and intervention. Several different groups of new mothers and fathers, serving deprived populations, were involved in PPI group discussions as described in the main study report.^20^ They discussed the interpretation and dissemination of the results and agreed that participants should be sent an easy‐to‐read study summary leaflet by post or email which they approved and has been done.

Ethical approval was received in November 2016 from South West—Cornwall and Plymouth Research Ethics Committee (16/SW/0336). The feasibility trial was also registered with the International Standard Randomised Controlled Trial Register (ISRCTN14760978).

## 3 RESULTS

Table 1 shows the characteristics of the women interviewed compared to all who received the ABA intervention. The women had an average age of 28 years, and their baby's ages at interview ranged from four to 21 weeks (mean 12 weeks). Participants' quotes are attributed to Site A or B, with their baby's age at interview (in weeks) and whether they were breastfed (bf) (including any breastfeeding) or formula‐fed (ff) at 8 weeks. Similarly, the IFHs (1‐13) were attributed to Site A (n = 6) or B (n = 7). Each theme reports the perspectives of women and the IFHs.

**Table 1:** Comparison of characteristics of all the women receiving the ABA intervention (n = 50) with those interviewed (n = 21)

Characteristic	All intervention women n = 50	Intervention women interviewed n = 21
Maternal age at baseline in years (mean and range)	28.6 y (18‐38)	28.9 y (19‐37)
Ethnicity—White British n (%)	43 (86.0%)	17 (81.0%)
Employment—paid work n (%)	40 (80.0%)	18 (85.7%)
Any breast‐feeding at 8 wk n (%)	24/48 (50.0%)	12/21 (57.1%)
Any breast‐feeding at 6 mo n (%)	18/39 (46.2%)	9/20 (45.0%)

Overall, women valued the opportunity of receiving support from someone with similar experiences and learning about what community assets were available. The volunteer supporters were excited by new opportunities to meet different women and provide support for several months, and the paid supporters appreciated the content but found that arranging visits to the women was difficult due to their workloads and distance to participants.

Five themes (Table 2) are reported that present the women's and IFHs' views of the ABA intervention components: ‘early opportunities for infant feeding conversations’, ‘mapping the friends and family tree’, ‘keeping in touch using proactive text messages’, ‘knowing about local groups and assets’ and ‘a woman‐centred approach’.

**Table 2:** Comparison of the ABA intervention themes between women and infant feeding helpers, illustrated with summary statements

Themes	Women	Volunteer supporters	Paid supporters
‘Early opportunities for infant feeding conversations’/continuity of helper	Good to have space to think about and discuss options. Helpful to contact the same person before and after the birth	Opportunity to see women before the birth to discuss feeding and support them proactively	Opportunity to discuss all feeding methods was valued. This is part of the paid job but not usually for women in this area
‘Mapping the friends and family tree’	Raising awareness of my available social support. Kept the map in my head	Enjoyed exploring all possible support with them. Kept it on my phone and referred to it in texts and calls	Mostly used it as a summary of our conversation and for data collection. Women did not want to keep the paper diagram
‘Keeping in touch using proactive texting’	She was encouraging and sent me positive messages every day	Liked being able to contact women proactively; they could answer when convenient for them. Increased contact was sometimes challenging for my family life	We struggled to fit this in during working hours. Some women were difficult to contact
’Knowing about local groups and assets’	They encouraged me to go and get support from other mothers. Did not know about the groups until my IFH told me about them	Women who would not normally come to the breastfeeding groups came along	The leaflet was useful to give them this information
‘Woman‐centred approach’ (using listening skills) not breastfeeding‐centred	Good to have time to talk about anything. They were reassuring, kind, supportive	New opportunity to talk to women antenatally and soon after birth	Some of ABA was already part of our job. Only some women wanted visits

### 3.1 Early opportunities for infant feeding conversations

Women recalled antenatal meetings with their IFHs as being a relaxed discussion and welcomed the opportunity to have a ‘chat’ about infant feeding whatever their preference. The ‘face‐to‐face’ element of the antenatal meeting was considered an important part of being able to develop a relationship with the IFH and encourage contact after their babies were born.

But just relieved once I had met her and I can put a face to the name, just gives you that reassurance again really that there's somebody there, you know who they are and she was really friendly and approachable as well, so it's nice, then I wouldn't feel like I'm texting her thinking what's she going to be like? So then didn't have a problem going away and thinking if I need to text her then I would. (P22 Site B, 14w, bf)

Most women found the antenatal meeting to be a positive experience ‘*it was really a good experience at that time*’ and the content useful, and they found it could stimulate interesting conversations about infant feeding.

Yeah it was good. I didn't think I had so many thoughts around breastfeeding as I did when she was starting to ask questions around it, I didn't think I had really thought about it as much as I obviously had, which was quite good. (P16 Site B, 10w, bf)

While for some, the meeting with their IFH resulted in them ‘*feeling a lot more positive’* about feeding, one woman who had been intending to formula feed described how it helped her to reconsider her feeding decision.

It made me rethink about breastfeeding… but having that chat with her it did re‐jog my memory there is another option sort of thing, yeah it did, definitely. (P6 Site A, 11w, started bf, ff by 8 weeks)

The antenatal meeting was less interesting to women when it seemed to be a fact‐giving exercise or when they had decided how they wanted to feed their baby and already felt well‐informed.

I think it was helpful, and it was nice to meet her, and nice to have the discussions and things, but yeah I'm not… I think I already knew that, I already knew what help I could have. (P1 Site A, 8w, ff)

IFHs in Site A were used to making postnatal visits to women at home but as ABA women were more remotely located, this created time and travel pressures, particularly for IFHs who relied on public transport. Despite these challenges, many perceived the antenatal meeting with women to be a positive addition.

It's not the areas that we usually cover, they're more central….if you're going to spend more time travelling it limits you to how many women you can see during that day… (IFH 2 and 4, Site A focus group)

…the support is when they need it, so it was knowing that it was there beforehand I think which does make a difference. (IFH 1 Site A interview)

The volunteer IFHs in Site B, however, who did not have such commitments and were local, were able to be more flexible in their contact with ABA women. They enjoyed the antenatal contact, despite it taking up time, and sometimes being emotionally challenging.

I think it's been positive; it's been a good experience. I have enjoyed it, but I have also found it quite time consuming and almost more emotionally involved than I thought it would be. (IFH 7 Site B)

### 3.2 ‘Mapping the friends and family tree’

Women provided mostly positive views about the mapping exercise to create their Infant Feeding Genogram (see Figure 1). Many found genogram completion to be useful as it helped them recognize how much support was available. Some women described the process as ‘*reassuring*’ as it reminded them how fortunate they were to have support.

She did a really useful thing actually, which was we did a map of people in my life that I could ask any help for feeding advice and things like that…and just it just made me rethink and evaluate how much I appreciate having some family closer by. (P23 Site B, 13w, bf)

However, a few women did not quite see the purpose of completing the genogram, perhaps because the IFHs had not explained it well enough and had difficulty discussing the concept in a meaningful way.

I just thought it was a bit weird that you asked about my family and my friends who had breastfed, I thought it was a bit what's that got to do with anything? But then thinking about it I was like well if they hadn't have breastfed and I hadn't have witnessed my bottle fed friends getting ill, maybe I wouldn't have breastfed, I don't know, you don't know. (P8 Site A, 6w, bf)

While most women did not use the physical paper copy of the genogram, they valued being able to retain a mental memory of the information.

I haven't really [referred back to the genogram]. I think it's put it in my mind once I'd seen it, but I don't need to look back on the paper, obviously knew who I had and just having contact with [helper] and my sister‐in‐law, and obviously my partner has been here all along. (P26 Site B, 10w, bf)

Some IFHs reported some less positive views about the genogram, particularly in Site A, where they felt that they usually covered this information with women, but doing an exercise on paper could be a ‘barrier’ to forming a relationship with them.

Some way down the line she will say I was breastfed, or partner was breastfed, it will just automatically come in anyway… So, it wasn't anything new that we were doing, but it's just this time we had to put it on a piece of paper. (IFH5 Site A)

IFHs completed genograms with all women seen antenatally (apart from one who declined due to family bereavement), but for some, it *‘took a while to get my head around it’* and appreciate its purpose. These IFHs felt they had used it more successfully with women supported later in the study.

I found that I did sort of refer back to it in my head a little bit like you said … and then I think for them again, especially the second, third and fourth ladies it just reaffirmed the support that they had. (IFH9 Site B)

Site A IFHs reported that the completed genogram did not feature during subsequent helper‐mother interactions and also that some women did not want to keep their completed diagrams. Some in Site B stated that while they had not used the paper copy, they still used the information as prompts during helper‐mother contacts and this helped to show personal interest and to feel more involved in a woman's life.

No, we didn't refer back to it, but it may have come up in a conversation, but we would never actually have gone with the physical genogram. (IFH3 Site A)

I would refer back to them and say is your sister [name] still popping round?….It certainly helped me feel like I was a little bit more involved in their actual lives rather than just them just being numbers on a page really. (IFH8 Site B)

### 3.3 ‘Keeping in touch using proactive texting’

Women seemed grateful for proactive contacts from IFHs, finding it reassuring that help was there if they needed it. Text messaging seemed to be women's most preferred and effective method of contact; mainly because it was *‘easy’*, they could respond in their own time and *‘have time to process it’*.

I preferred that. I didn't really have much energy to form proper sentences at that point… so texting was much better. (P26 Site B, 10w, bf)

A text message you can answer in your own time, that's the positive of a text message, rather than a phone call that you have to either miss or answer straight away, you can answer it in your own leisurely fashion. (P8 Site A, 4w, bf)

Failure to respond to IFH text messages was often due to the demands of caring for their new baby or not needing help, rather than not wanting to be contacted. One woman described how receiving texts gave her ‘permission’ to continue seeking advice for longer than if she'd had to instigate the contacts herself.

If they hadn't offered their help, I'm not sure how good I would have been about asking for help… I suppose I kept feeling like I should be beyond the stage of needing their help… but with them asking how I was it gave me permission. (P4 Site A, 9w, bf)

IFHs made positive comments about the schedule of suggested contact times as *‘you could see what you had to do’.* The smaller caseloads at Site B meant providing the agreed number of contacts was manageable, although one reported that it was challenging to manage a home/life balance. They made all their early postnatal contacts by phone or text until they were able to meet up at local support groups.

When you've got your own children, it's trying to fit it all in, and I think there might have been a few times where I missed by a few days. (IFH12 Site B)

However, IFHs in Site A found fitting ABA postnatal contacts around their busy working schedules more difficult.

It was sad that the women didn't actually respond back, so it was very difficult to get hold of them, especially getting to know them … that was quite difficult, they didn't really engage. (IFH4 Site A)

IFHs negotiated the frequency and method of contact with each woman and encouraged women to contact them and seek out help as needed. Sometimes, this meant that they could reduce contact based on their assessment *‘she is doing really well’* or providing additional help as it was felt to be *‘the best thing to do’*.

I made it clear that they could text me whenever they wanted, and I would get back to them as soon as I could. (IFH7 Site B)

IFHs in both sites expressed some concerns that the frequency of proactive contacts may be construed as ‘*hassling*’, particularly when there was a lack of response, so making them unsure how to proceed. Sometimes, this reluctance to be proactive resulted in reducing the number of contacts to give the mother ‘*a bit of space*’, indicative of a sensitive woman‐based approach.

Didn't want to keep phoning them when they've just had a baby so if they were happy to text or if they wanted to call, whichever they wanted basically, just worked it round them. (IFH 1 Site A)

### 3.4 ‘Knowing about local groups and assets’

Women provided positive comments about the assets leaflet which contained information about local groups, websites and phone lines for support. They mentioned being aware of some, but not all, of the resources listed, and that there was more support available than they had expected. One woman reflected that while she had already been thinking about going to local groups, the assets leaflet helped to remind her where and when these activities were, and she particularly valued the offer from the IFH to accompany her.

I think we were surprised about the amount of clinics that there were, .. here, there and everywhere, and that run most days. (P2 Site A, 21w, ff)

She said if I wanted to, she would meet me at them and to come with me. She went through all the different groups and stuff…so that was helpful. (P21 Site B, 12w, ff)

Women reported that they used the resources described in the leaflet including attending breastfeeding groups, accessing websites or joining Facebook groups. One mentioned that she kept the leaflet *‘to hand’* for ease of access, and how it was a useful reference to look for information and answers *‘should she need it’*:

I knew that if I needed help, I could access it, so I suppose that was in the back of my mind, it was like well at least it's there. (P1 Site A, 6w, ff)

Yes, I have, it's somewhere, I think it's in the changing bag actually. I try to keep it to hand, and yeah just spent probably many a late night at first going through it looking on websites, is this normal? (P22 Site B, 14w, bf)

While some of the women did not access any of the resources provided, this was often because they did not require additional help, rather than the quality or availability of support. The only negative comment given concerning the leaflet was about the amount of printed information that pregnant women/new mothers receive, with the assets leaflet just one more piece of paper to keep track of.

Problems in getting to groups in the early weeks, particularly following caesarean section, were mentioned which potentially prevented these women getting the support they needed.

However, one woman who attended a breastfeeding group said her partner had encouraged her to attend, for reasons unrelated to infant feeding. She described benefits of the group as giving opportunities to socialize with other mothers and enhancing positive feelings towards breastfeeding.

I think he [partner] was very keen that I needed to get out of the house with her on my own before he went back to work. It's just nice to speak to other women, and I've always felt more positive towards the feeding after going to the group. (P19 Site B, 16w, bf)

IFHs at both sites confirmed women's use of the assets leaflet, including accessing antenatal group sessions, or attendance at breastfeeding groups.

When I rang her… she says that she's been to [a group] “It's local to me and I've been to that one and it's quite good and I'll go again every week.” (IFH2 Site A)

IFHs and women in Site B considered that having the antenatal meeting in the same venue (ie Children's Centre) as the local breastfeeding group was helpful in encouraging them to access group‐based support postnatally.

At the meeting, because we were obviously in the Children's Centre, I showed both the ladies where the breastfeeding group would take place, they knew the building, and I think that helped when they did come along [postnatally]. (IFH7 Site B)

### 3.5 ‘A woman‐centred approach’

A key feature of the ABA intervention was offering support using a ‘woman‐centred approach’ rather than having a breastfeeding‐centred focus in all discussions.

Women mostly felt that this had been achieved when they described being reassured that they knew where to go for appropriate advice and support, not feeling that they were being pressured to breastfeed and receiving positive feedback and encouragement from their IFH.

I said that I wanted to bottle feed, but if I did breastfeed probably mixed feed so that my husband could feed her as well, and it was really nice to talk to her actually because normally if you talk to somebody about what are you going to do they pound on, are you going to breastfeed sort of thing, but she was really whatever suits you is best, not breast is best or not bottle is best, what suits you. I suppose she was saying keep an open mind but because she was so neutral to both bottle and breastfeeding, I didn't really feel pressured by her…It made me rethink about breastfeeding again. (P6 Site A, 11w, ff)

She's always come from quite a non‐biased opinion, so she's always given me, this is what this is, she's not ever been this is what I think, this is what you should do, she's always been very open and this is what can happen, and always been so lovely with you're doing so well, you're doing so brilliant, because especially in the early days you doubt yourself and you feel am I doing it right? and is he getting enough? (P23 Site B, 13w, bf)

IFHs also reported that they understood and tried to use a woman‐centred approach when reflecting on the training and in describing some of their early contacts with women.

It [training] was all good, like [name] said the role playing, because the discussion around the mum‐centred bit rather than being breastfeeding centred, just trying to shift gear a little bit and have different mind‐set about that…. the emphasis just being on building a relationship was useful. (IFH11 Site B)

What we tended to do is that we made sure that when we did make contact with them, every time that we're from infant feeding so they didn't think that… because a lot of times they had the perception of breastfeeding, they think oh they're going to be there to pressure them into doing it, and what we said a lot we're from the infant feeding, ABA infant feeding, and we're there to support you however you want to feed. (IFH3 Site A)

My first lady… we did text for quite a long time not necessarily about baby stuff but about her being ill and that, probably longer than the two or three weeks just because I thought we were getting on quite well… I just felt like that was the best thing to do really, I didn't want to just abandon her when she was mid‐treatment, so I followed it through. (IFH8 Site B)

## 4 DISCUSSION

This qualitative study explores women's and IFHs' views (paid peers and volunteers) of the different ABA intervention components. Overall, women were positive about the antenatal meeting in terms of early opportunities to discuss infant feeding and how it facilitated ongoing regular woman‐IFH text‐based communications. Women found mapping their network of support to be helpful and reassuring, and the assets leaflet stimulated them to use available community assets.

While IFHs were generally positive about the different ABA components, the diversity of local neighbourhoods (urban vs more rural) and flexibility in supporter time (restricted paid hours vs flexible volunteers) had some influence on the ability of the helpers to embrace the intervention. These differences influenced the approach of some IFHs to the ABA intervention and their engagement with the participants.

### 4.1 Early and proactive support

Proactive support has been reported by others to be effective in increasing breastfeeding rates.^8^, ^9^, ^10^, ^21^ Continuity of targeted peer support with an antenatal visit and postnatal support from the same local supporter has been shown to be associated with psychosocial benefits for mothers, health professionals and peer supporters.^11^, ^22^ Proactive women‐centred contact providing continuity of care from pregnancy to several weeks after birth was also valued by women in a small study,^9^ and very early postnatal support has been reported as an important factor for effective breastfeeding support.^12^

### 4.2 Assets‐based approaches incorporating the infant feeding genogram

The assets‐based approach via use of the genogram and the assets leaflet were highly valued features of the ABA intervention. Such approaches are in line with sustainable models of community development via extending networks and building social capital.^23^ Assets‐based approaches have been used in a range of public health studies.^15^, ^24^ For breastfeeding, these could focus on both intrinsic personal resources such as self‐efficacy in relation to infant feeding^25^ and motivation and drive to maintain feeding,^26^ and external resources such as family and friends; wider social networks of women who have breastfed; and community assets such as children's centres, mother and baby or breasteeding groups, and local breasteeding peer supporters.^27^ The theory of change approach for assets‐based working proceeds through four stages: (a) reframing thinking, goals and outcomes; (b) recognizing the assets available to achieve the change; (c) mobilizing assets for a purpose; and (d) co‐producing outcomes.^28^ The discussion with the IFH, with prompting via the infant feeding genogram and assets leaflet, facilitated movement through these stages towards a co‐produced map of their existing assets landscape, which helped women restructure their social environment and increase their personal and external resources to support feeding their baby.

The Infant Feeding Genogram was developed in 2014 as part of a study exploring how women who were the first to breasteed in a family made sense of their decisions.^16^ Our study is the first to explore its wider acceptability, and this is further analysed by Thomson et al.^29^ The genogram gives detailed information about the family structure and the interactions between generations, but it does not show relationships with a wider social group. The way that the IFHs used the genogram with women might be better described as a sociogram, another family therapist tool or a mixture of the two, giving a picture of the many supportive relationships available to women.^30^ Strengthening the use of the modified genogram in a refined ABA intervention would help IFHs understand the processes involved.

### 4.3 Peer supporters using behaviour change techniques

Using peer supporters to provide social support and restructuring the social environment with a woman‐centred approach through encouragement and advice has been recommended by others. A meta‐synthesis of women's experiences and perceptions of breasteeding support found that a person‐centred approach was more acceptable than breasteeding‐focussed discussions.^6^ Women in other studies have welcomed a peer supporter approach that helped them mobilize external and personal resources to achieve their breastfeeding goals through words of praise and reassurance.^5^ A recent feasibility study using motivational interviewing techniques as their peer supporter intervention (Mam‐Kind) reported that supporters found it quite challenging to move from an ‘expert‐by‐experience’ role to a more collaborative approach when giving information.^31^ A similar challenge was also implied in our study by some IFHs (Site A) who felt that many ABA intervention components, such as being women‐centred, were already part of their role, and some failed to perceive the value of co‐creating the genogram.

Other studies have examined the influences of significant others on women's feeding behaviour and emphasized the importance of holistic family‐centred approaches to supporting women.^32^ Similarly, helping women to become familiar antenatally with the venues where postnatal groups are held to facilitate return after birth, with someone who can introduce them to a group on the first occasion (such as an IFH), has been shown to influence why interventions work in some places and not others.^4^, ^27^, ^33^

We will use the findings from this study to modify the design of the information materials for women and training given to IFHs in our future trial. We will provide more explanation of how to incorporate and deliver the behaviour change techniques of restructuring the social environment and providing social support using an assets‐based approach and more practical discussion about how to deliver the assets materials.

### 4.4 Strengths and limitations

The study strengths include exploring a novel assets‐based approach to delivering infant feeding support and including all women regardless of feeding intention. We compared the perspectives of IFHs and women who received the ABA intervention and included two different sites with different delivery models (paid workers and volunteers).

We achieved rigour in this study by use of detailed data analysis, undertaken by multiple researchers and analytical decisions being shared with all team members to achieve credibility. The researchers have a range of health‐related backgrounds with prior experience of evaluating peer support. None of them were involved in direct delivery of the intervention, and all were involved in the data analysis. We have included a wide range of quotes, from different individuals across the two sites to illustrate the final interpretations. All quotes are supported by demographic details to enhance transferability of the findings.

Although the use of PPI within the ABA study provided us with a vital user perspective, it was challenging to sustain relationships with some. Pregnancy and caring for young children take up a relatively short period of women's lives, and inevitably, they move on by returning to full‐time work or being involved with school activities, which can make it difficult to have continuity with PPI contributors.

Limitations include our sample of ABA intervention women interviewed; all returned the 8‐week questionnaire, and so the views of the nine questionnaire non‐responders in the trial are unknown. A slightly higher proportion of women interviewed were breastfeeding at 8 weeks than for the whole intervention group, but otherwise those interviewed were similar to the women in the trial.

## 5 CONCLUSIONS

Women who received the ABA intervention and paid and volunteer IFHs who delivered it welcomed this approach, despite some challenges in its delivery. The components of the intervention, including the infant feeding genogram and local assets information, were perceived to be useful in exploring and highlighting available sources of help that women could draw on for advice and support.

This proactive community assets‐based approach with a woman‐centred focus is a promising intervention that warrants further research to explore its effect on infant feeding outcomes.

## CONFLICT OF INTEREST

JI, GT, DJ, JC, PH and SD have no conflicts of interest. KJ is part‐funded by the National Institute for Health Research (NIHR) Collaboration for Leadership and Applied Health Research and Care. HT is a Trustee for NCT Charity, which provides infant feeding support, and a breastfeeding counsellor for the same organization.

## Supporting information

Supinfo

## Data Availability

The data that support the findings of this study are available from the corresponding author upon reasonable request.
