# Supplementary material for: Women's and peer supporters' experiences of an assets‐based peer support intervention for increasing breastfeeding initiation and continuation: A qualitative study
Source: Health Expect. 2020 Mar 21;23(3):622–31. doi: 10.1111/hex.13042 (PMC7321743; doi:10.1111/hex.13042)
Supplement: Supplementary file 1 — Supinfo [file HEX-23-622-s001.pdf]

**INTERVIEW TOPIC GUIDE FOR WOMEN**

**This is the starting topic guide; questions and prompts will be developed as interviews are undertaken to incorporate any important areas emerging from the interviews.**

1. Experience of feeding their baby.

- How have you been getting on with feeding your baby?
- Have you experienced any difficulties?
    - What did you do?
    - Who did you go to for help?
- Before you gave birth, did you have plans for how you would feed your baby?

2. Experiences of the **ABA intervention** (taking account of feeding choices):

- Tell me about the first time you met the ABA infant feeding team member?
    - How would you describe your relationship with the feeding helper?
- What did you think about the texts you received before your baby was born?
    - To what extent did the texts encourage you to access wider support?
    - What different forms of support did you access?
    - What was your experience of these different forms of support?
- What about after you gave birth, did you let the ABA infant feeding team know that you were going home?
    - How soon did you talk to the feeding helper?
    - Was it the same person you met at the start?
- How did you feel about the calls and texts after you gave birth? (explore how often, how long they lasted, were they sufficient to meet needs?)
- Can you tell me about the infant feeding support you have received from your partner, friends and family or other community groups?
    - To what extent did the infant feeding helper encourage you to access this support?
- What (if anything) did you find helpful about the ABA feeding team support?
    - To what extent did the ABA feeding support influence your infant feeding experiences?
- What (if anything) did you find unhelpful about the ABA feeding team support?
- What would you change about the ABA feeding team support?
- Do you have any other issues or views you wish to share about your experience of the ABA feeding team?

**INTERVIEW TOPIC GUIDE FOR ABA INFANT FEEDING TEAM MEMBERS**

This is the starting topic guide; questions and prompts will be developed as interviews/focus groups are undertaken to incorporate any important themes emerging.

1. *Overall perceptions and experiences of the ABA intervention*

- What are your thoughts about the ABA intervention?
  - Were there any particular elements that you liked or disliked?
  - Where they are elements that were difficult to deliver?
  - What were the challenges in delivering the intervention?
  - What worked well?
  - How would you change it?
  - Overall, how would you describe your experience of delivering the intervention?

2. *Delivering the intervention & Impact on women*

- How soon did you manage to contact women after they were discharged from hospital?
- Can you tell me about difficulties or challenges in delivering the intervention as planned?
  - Were there any difficulties in contacting women (such as sending texts)?
  - Can you tell me about any changes that you made when delivering the intervention?
    - Why did you make these changes?
- What impact do you think the intervention had for women in terms of them drawing on support from partners, friends and family or community groups?
  - How is this different from the support you previously provided?
  - How did you cope with the increased demands?
- What do you think women felt about the intervention?
- What difference (if any) do you think the intervention made to women's infant feeding experiences?

3. *Coordinating the ABA intervention*

- Can you tell me about any challenges in delivering the intervention between ABA team members?
  - How did you resolve them?

Did it have any costs in terms of working longer hours, need for childcare, telephone calls?

4. *Experience and adequacy of the training*

- Can you tell me about your experience of the training?
  - What was good about it?
  - What did you learn that was new?
  - What was not so good?
- Can you tell me about any practical issues or challenges faced during the training (e.g. timing, duration, location)?
- How has the training changed your practice?
- What would you change about the training?

5. *Additional comments*

   Do you have any other issues or views you wish to share about the ABA intervention?
